# “*We Speak the Same Language, but They Don't Understand Us*.” Use and Abuse of Culturalism in Medical Care for Central American Migrants in Transit Through Mexico

**DOI:** 10.3389/fpubh.2022.880171

**Published:** 2022-06-14

**Authors:** Philippe Stoesslé

**Affiliations:** ^1^Departamento de Ciencias Sociales, Universidad de Monterrey, San Pedro Garza García, Mexico; ^2^Université Paris Cité, Unité de Recherche Migrations et Société, Paris, France; ^3^French Collaborative Institute on Migration, Paris, France

**Keywords:** culturalism, stereotypes, naturalization, healthcare, migrants, Mexico, discrimination

## Abstract

This article deals with cultural stereotypes toward Central American migrants in the Mexican healthcare system, which lead to the naturalization of the supposed cultural characteristics of these new users. Based on 21 interviews of health and administrative staff in the state of Nuevo Leon (northeastern Mexico), it shows the first recourse to culturalist explanations to describe any phenomenon related to migrants' health. According to this perspective, the health of migrants, their relation to illness, and their patterns of seeking healthcare would be mainly determined by characteristic cultural traits, which justify their penurious attendance at health centers, and their low adherence to treatments. The culturalist explanation of migrants' health behaviors may influence the care they receive, as well as their adherence to treatment, which ultimately reinforces the health inequalities initially highlighted. This culturalist excess is partly explained by the incorrect understanding of the directives of health authorities in favor of the integration of an intercultural perspective in healthcare. Despite some ongoing training in this area, it does not seem sufficient to correct this situation effectively.

## Introduction

This article addresses one of the cross-cutting issues related to the difficulties of access to healthcare for Central American migrants in Mexico: the inadequate reception of these “new users” in the healthcare system, and by healthcare personnel. The latter incorrectly represent migrants, often based on abusive “cultural” stereotypes.

Although this subject bears one of the deepest roots of the health inequalities that migrants face during their journey to the United States, it is rarely addressed in scientific studies on this area (this phenomenon is much more studied in other parts of the world). In addition, the cultural awareness of healthcare personnel is a very present topic in the field of health education, and the practice of care from medical schools to healthcare facilities. However, the existence of misrepresentations does not seem to be a concern for the authorities.

Even so, administrative and healthcare workers are not exempt from these pitfalls because their initial training does not necessarily provide them with the tools to identify and dismantle them. This phenomenon, for example, was identified by Paz and colleagues (1) in their study on migration and health on Mexico's southern border but has never been studied in the northern part of the country. In the state of Nuevo Leon (Mexican northeastern border state with Texas), the stigmatizing representations that are widely disseminated among the population also rub off on healthcare workers. This idea is hardly surprising, as caregivers exhibit the same levels of implicitly biased views as the general population (2).

The primary objective of this research is to question the appeal for “culture”^1^ by the agents of the Mexican health system, to explain the limited access to health services experienced by migrants in transit through Mexico (3). According to this perspective, the health of migrants, their relation to illness, and their patterns of seeking healthcare, would be mainly determined by characteristic cultural traits, which justify their penurious attendance at health centers, and their low adherence to treatments.

Given the foregoing, we aim to clarify the perception that health system agents have of the link between health and culture, specifically in Central American irregular migrants. Are their health behaviors culturally marked? Why do health professionals' resort to this explanation, and what validity does this “culturalist”^2^ analysis possess? How do health professionals integrate these cultural considerations into their care? Likewise, the production of mechanisms for the differentiation of migrants and the naturalization of their supposed differences in the Mexican health system is analyzed, by integrating particularly the contributions of French and Canadian sociology.

We present here the preliminary results of a fieldwork conducted in the ongoing research project of the *Universidad de Monterrey*, “Perception, integration, and discrimination toward migrants in Nuevo Leon,” [registry number FINV21090]. The project focuses specifically on the factors that promote and hinder access to health for Central American migrants (mainly native to Guatemala, El Salvador, and Honduras) in irregular transit through the city of Monterrey, Nuevo Leon.

The research is based on formal and informal interviews conducted between November 2017 and May 2019 in four urban medical centers (CSU in Spanish, “Centros de salud urbanos”), four public and private hospitals, one registration center to the *Seguro Popular*^3^, *three* municipal public health departments, and at the State Human Rights Commission. In the 21 formal semi-structured interviews we conducted on migrants' access to health, medical, and administrative staff of the healthcare system in Nuevo Leon, as well as public health officers, evoked their representations, experiences, and practices with migrants.

The study respondents were selected according to non-probabilistic sampling and by convenience. These are the people who agreed to participate, although an attempt was made to reach a diversity of agents who could provide a maximum of information regarding our main objective. The number of interviews was decided by the saturation of the information gathered, when no more new data appeared in the new interviews.

The list of participants, as well as their function, is detailed in Table 1.

**Table 1:** List of participants.

	**Organization**	**Position**
1	CSU 1	General practitioner
2	CSU 2	General practitioner
3	CSU 3	Psychologist
4	CSU 4	Social work manager
5	CSU 4	Director
6	Private hospital 1	Administrative manager
7	Private hospital 2	Administrative official
8	Private hospital 2	Charge nurse
9	Private hospital 2	General practitioner
10	Public hospital 1	Social worker
11	Public hospital 1	Nurse
12	Public hospital 1	General practitioner
13	Public hospital 2	Nurse
14	Public hospital 2	Specialty doctor
15	Public hospital 2	Social Work Officer
16	Public hospital 2	Charge nurse
17	Registration center to the Seguro Popular	Administrative officer
18	Municipal Public Health Department 1	Director of Public Health
19	Municipal Public Health Department 1	Health Promotion Officer
20	Municipal Public Health Department 2	Director of Public Health
21	State Human Rights Commission	Director of Human Rights Promotion

Obtaining these interviews was not easy, as entering the clinical space is difficult for those who are not part of it. It is not easy to bring in an outside perspective in a milieu where sociologists are perceived as unnecessarily “critical” (in the destructive sense of the word), often illegitimate, and with concerns that rarely fall within the scope of “allopathic” physicians concerns.

The healthcare world is generally reluctant to accept third-party mediation, let alone to address socially sensitive issues. Some participants tried to dodge questions that they considered uncomfortable, and it is worth noting that several public health officials from the Nuevo Leon government never responded to our interview requests (by phone or e-mail). A senior official in charge of migrant health in the Ministry of Health stopped all communication the day he received the list of questions that needed to be answered.

On the contrary, a “snowball effect” worked in parallel, especially with agents located at the bottom of the hierarchy, who are often sensitive to the abuse suffered by undocumented migrants. This empathy was particularly present among social workers, who were by far the most helpful in helping us to contact both colleagues and managers.

Once the appointments were obtained, the interviews made possible to reconstruct, from the perspective of the health workers, their interpretation of their experiences and their relationships - sometimes conflicting - with the migrants. The respondents were asked about their knowledge and experience in the access to health services for irregular migrants, the existence or not of specificities for this population, and about the culture of migrant patients in their passage through the health system. Finally, we collected information about their education and professional training on interculturality, which turned out to be a very enriching issue for the analysis and contextualization of the topic. To guarantee absolute confidentiality and protect the strict anonymity of participants and informants, we decided to conceal their sociodemographic information, resort to a masculine-neutral gender when we present their testimonies and exclude details that would allow the identification of the informants or their institution of belonging.

Even if the empirical work in this research predates the COVID-19 pandemic, it is undeniable that the issue presented here takes on even greater relevance in the face of the current health emergency affecting the most underserved populations. Indeed, in the context of the COVID-19 pandemic, the factors that previously endangered the lives of these populations (such as insecure travel conditions, social marginalization, overexposure to organized crime, dangerous means and routes of transportation, extreme weather conditions, and limited protection from the State; despite the obligations under national and international law) have been exacerbated by the lack of opportunities and reduced support networks. It has also been shown that migrants are prone to be the targeted by COVID-19 related stigma and discrimination (4).

Besides, cultural essentialism is a constant behavior in history. Sanitary crises often reinforce these thoughts, as history shows. From the Black Death of 1,346 to the COVID-19 crisis (passing through AIDS, the Influenza AH1N1 pandemic in 2009, and the Ebola epidemic of 2014), migrants are stigmatized as the “*carriers of disease*” (5). Paradoxically, one of the most vulnerable groups to the disease finds itself responsible for its expansion (6), and stigma tends to fall most harshly on communities living in a situation of constant mobility (7). Another clear example is the case of the Spanish Influenza of 1918, during which Spanish immigrants suffered violence and discrimination as they were called the “source” of the virus because they had high rates of contagion, even though the disease occurred during the First World War in a camp in Kansas, USA (8).

After initially discussing the injunctions that caregivers receive to treat patients equally, the second subsection turns to the use of culture as an explanation of health behaviors. The connection between culture and health and their incorporation into the guiding principles of the Mexican health system is then discussed, before concluding with an analysis of the culturalist discourses that essentialize migrants and naturalize their “differences.”

## Equal Treatment As the Guiding Axis of Social Services

In Mexico and the rest of the world, migrants are often among the groups that face the most obstacles in accessing health (9, 10). Aware of this and other structural inequalities, the Ministry of Health in Mexico introduced the principle of equal treatment in access to health as the basis of its vision, to achieve “*a National Universal Health System, equitable, comprehensive, sustainable, effective and quality, with particular approach on population groups living in vulnerable conditions”* (11).

In practice, administrative and health professionals are the constant target of institutional communication (from the universities, their institutions of belonging – health centers, hospitals –, the professional associations, and especially the Ministry of Health) whose mantra is to “humanize” the treatment of patients. This equality entails a principle of non-discrimination, which prohibits any differential treatment in health institutions. The “equal treatment” injunctions are thus everywhere, though its definition is as confused as its implementation.

The attachment to this almost sacred principle stands out in our fieldwork. The health workforce altogether demonstrates their firm intention to not derogate from it. In this regard, all claimed to be respectful and guarantee the same quality of care to all individuals without any distinction, completely erasing any difference between migrant and non-migrant patients: “*because they are people, migrants deserve respect* […]. *We must take care of them with humanity and mercy*” (general practitioner, CSU). A specialty doctor from a public hospital points out: “*Ethics must prevail at all times* […] *we must humanize care* […] *and adhere to the policy of zero rejection. It is a state duty to protect the integrity of each and every person within the country*”. Finally, an administrative manager of a private hospital testified that “*both the poorest and the richest are respected*, […] *we respect human rights*”.

This concern for the health of all individuals extends and reinforces when we question the situation of migrants in Nuevo Leon: “*We all know what they go through and what they experience, it's impossible not to be sensitive to that*” (nurse 1, public hospital). “*They have no privileges, and we must help them*” (nurse 2, public hospital). “*I believe that we are somehow allies so that they face the challenges of daily life* […] *it is also our interest that they heal correctly*” (specialty doctor, public hospital).

Certain professions seem to have a special sensitivity, from the initial training of their members. This is how the person directing social work in a public hospital explains: “*in professional training, that is palpable since it is in your education as a professional; interculturality, non-judgment, accepting the human being, how the human being is, with all his beliefs, with all his culture, with everything that entails*” (author's emphasis).

Superficially, the question of culturalist misrepresentations does not seem to arise for healthcare providers. And when it is asked, it is immediately dismissed with a discourse based on human rights and the universalist values of the health system. Yet, there is no reason a priori why the individuals who drive this system should not share any of the prejudices and stereotypes rooted in Nuevo Leon and Mexico.

It is a fact that there is no reason to be skeptical about the genuine kindliness, empathic intentions, and devotion of the interviewees to the principles of equal treatment and non-discrimination, although when questioning them about the challenges of caring for people in a mobility situation, specific comments including aspects of cultural categorization begin to come out to the surface.

Hence, a public health officer at the municipal level mentions that “*sometimes, the cultural habits of people who come to the country are already very rooted in, for example, they are used to being treated with alternative methods*”. A colleague of his, in charge of health promotion, explains: “*they are deeply rooted in their habits and customs in their ways of life, where they come from* […] *it is no easy, but it is about promoting in them, as in all people, a new culture of health so that they go to their health unit*.”

In calling for “*a new culture of health*”, this senior officer calls for a substitution of what he perceives as meaningless “habits and customs” that would impede a “modern” process of care. Central American migrants would be fervent users of traditional medicines. According to this simplistic causal imputation, their “magical” beliefs, rooted and transmitted within their families for generations, like an inherited genetic heritage, are detrimental to their health behaviors.

It is not unusual to see interculturality caricatured in the folkloric and fixist practices of these “others”, who would be culturally over-determined. Pretceille (12) exposes that “*any excessive focus on the specificities of others leads to exoticism, to a fossilization of cultural practices with a consequent accentuation, conscious or not, of stereotypes and prejudices*” (p. 257).

Thereby, references gradually appear to categories such as the “culture,” “customs,” “habits,” or “ways of life” of migrants, that according to those who evoke them, must be included to improve the efficiency of care and adapt them to the presumed continuous mobility of patients. A “*presumption of difference*” then emerges (13) (p. 6), along with the first traces of differentiation between migrants and healthcare providers, but also between migrant and non-migrant patients: “*You have to see how they think because in a condition of this type it can get complicated in a person if you do not identify it in time* […] *it implies more risk for the same person*” (general practitioner, CSU, alluding to a Honduran patient with a possible virus).

## Culture As An Interpretative Key to Health Behaviors

Far from perceiving their differences in the other's culture, caregivers, therefore, tend to identify “differences” more easily than “similarities.” However, during an intercultural encounter, if there is not a modification of one's orientations – a “*decentration*”, as Porcher calls it (14) (p. 4) – the tendency will be to “*transform the other into a thing*”, as Sartre says (14) (p.5), and to create “*a planetary monoculture*” (Levi-Strauss, quoted by Porcher) (14) (p.6).

This over-interpretation of differences about culture leads to a categorization of populations (13). Previous studies have shown that healthcare personnel sometimes make this “cultural explanation” when faced with “atypical” patients, who are unfamiliar with the logic of seeking care. Cognet and Montgomery (15) point out that “*in health and social work organizations*”, the supposed “*cultural difference of users ‘of immigrant origin*”' is generally formulated in terms of “*a problem or obstacle to care and services*” (p. 7, 8).

In a complementary way, Cognet et al. (16) emphasize that:

(… ) this can result in a lack of care (from unequal relations causing tensions in the therapeutic relationship that will affect the process and possibly lead to a breakdown in care and treatment to refusal of care), differentiated care in the name of culture (13, 15, 17–22) or by an over-investment linked to cultural, psychological, physiological or genetic presuppositions, due to “ethnic” and/or “racial” classification (17, 23–26). And finally, by a lack of consideration of other factors (such as socioeconomic, environmental, etc.) that may affect health status, the evolution of health problems, responses to treatment and care, and their follow-up. This does not preclude the possibility of undisguised pro-nationalist, xenophobic, racist, and anti-Semitic expressions and sentiments. (p. 12)

The stakes are high because undocumented migrants face difficulties in exercising their right to health, since “*Civil society organizations report* […] *situations in which the lack of awareness of health officials regarding the situation of migrants is evident*” (27) (p. 270).

The WHO confirms the prevalence of this problematic situation among service providers (28). The Global Knowledge Partnership on Migration and Development (29) agrees that “*a common obstacle that migrants face concerning equal access to or enjoyment of health services is the lack of intercultural health policies and practices*” (p. 25), and this situation has become one of the “*common barriers to healthcare encountered by migrants*” (30) (p. 15) during the COVID-19 pandemic.

The International Organization for Migration (31) adds that:

The attitude of health professionals and others working in health facilities, as well as the quality of education they have received to sensitize them to the reality of migration, largely determine whether migrants will be able to use health services meaningfully'. (p. 50)

The main problem caused by this lack of sensitivity and training to the othering of migrants is that “*in the field of health, it* [culture] *becomes a real outlet for the problems faced by all those involved (doctors, nurses, social workers, etc.) in dealing with otherness*” (p. 43) (15). However, it should be recognized at the outset that integrating the cultural dimension into care is not an easy task, as Martine Pretceille identifies:

Between the “cultural zero,” that is to say the ignorance or the negation of the cultural dimension, and the “all cultural,” that is to say an overvaluation of the culture as a factor determining the behaviors, the margin of maneuver is narrow. Thus, the relatively recent recognition of cultures tends to a “dictatorship” of the cultural by reduction of the individual to his cultural belonging and by an overvaluation of the cultural dimension which leads to a culturalist and differentialist drift (12). (p. 252)

Let us also clear up an initial possible misunderstanding: negative caregiver attitudes and the resulting distinct behaviors are not exclusive to Nuevo Leon or Mexico. Similar situations have been documented toward Mexicans in the United States. For example, Holmes' work identifies a link between prejudice – sometimes unintentional – related to migratory irregularity and health discrimination against indigenous Triqui people while in the United States (in California and Washington State) (32).

Although health services have traditionally been criticized for being “doctor-centered” - that is, organized asymmetrically and “vertically,” where the doctor insensitively treats all bodies in a merely “organic” way, and without including the psychosocial and socio-cultural dimension of patients (33, 34), the consideration on the use of culture to understand a patient's health situation is not new.

Since the second half of the last century, Parsons (35) analyzes the health sector as a social subsystem and reflects on the behavior of sick people, coining the notion of “role of the sick” (“*sick role*”) to describe the rules of access to the status of “sick” through medical consultation. Subsequently, Mechanic (36) studies the cognitive aspects (how the person who suffers a symptom is perceived) and the taken actions (health behavior; seeking professional help, for example), coming to define an “*illness behavior*”. In 1966, Zola (37) reflects on the relationship between symptoms and culture, demonstrating that there are cultural expressions of symptoms and even complaints expressed by patients.

Later, Paul (38) identifies three reasons for the misunderstandings that can arise in a healthy interaction. First, is the assumption that the “others” have “unusual and peculiar” customs and beliefs. Subsequently, the perception of a superiority complex in ideas and methods (ethnocultural), and that “the others” are the ones who must rise to their level. Finally, the consideration of customs and beliefs as isolated from one another, rather than seeing them as a system of thinking together in which every cultural aspect is related and affects others.

Gradually, culture is positioned as a crucial explanation to health behaviors, especially the attitudes and subsequent actions of patients in their insertion into the health system. Up to the present, many studies have been published on the subject, such as Lebreuilly et al. (39) who explain the role of culture in the expression of pain.

These and other studies elucidate that the complexity of the illness process, and the interaction with a health professional is partially determined by the education and culture of all agents, in addition to their characteristics (such as their physiological and emotional health situation). However, it does not mean that in the case of migrants, the culture “of the country of origin” can be used in a deterministic manner, as a unique explanation of their health behaviors. As explained precisely by the head of the nursing department of a hospital: “*they're human beings like anyone […], everyone has their own culture and not everyone behaves the same just because they come from the same country*”.

Although there are shared representations and practices within the same social group, migrants passing through Nuevo León come from a myriad of different social groups. It would be a mistake to conceive the “culture of origin” as unique and consistent, denying in passing all unique characteristics, chosen identities and individual trajectories.

## The Promotion of Interculturality and Its Contradictory Enforcement

To avoid falling into such excesses, the focus has been placed on health as a common element between the diverse cultures and the sociocultural particularities of each patient. Indeed, disease behaviors are not universal, as they change in people, since illness occurs in different forms and with different symptoms, in addition to influencing individual perception in its definition and interpretation.

In this way, health authorities have promoted intercultural health, defined as “*the set of actions and policies that recognize and incorporate the culture of the user in the healthcare process”* (40) (p. 1061). To accomplish the objective, several pieces of training have been offered in which health professionals learn to adapt their attitudes and behaviors to the diversity of patients they serve, to become culturally competent (41, 42).

This “competence” reflects in recognizing and incorporating at all levels of its practice the importance of culture, the evaluation of cross-cultural relations and monitoring of dynamics resulting from cultural differences, and the adaptation of services to meet the specific needs of each patient (43, 44). Bernales et al. identify “*cultural competence*” as an “*urgent need*” for healthcare workers (45).

Its relevance is greater, since Antezana and Osmar (46) insist that the lack of intercultural competition can produce cultural clashes and in extreme cases, confrontations between patients and health professionals. Subsequently, Spector (47) explains that conflict often arises when health providers judge beliefs with their social norms and the health practices of others outside their social group.

In Mexico, health professionals are under strong injunctions to “take into account the culture of the patients.” The directives of the health authorities insist on interculturality, which is also present in the public policies of “migratory governance.” For example, the most complete of these – the *Programa Especial de Migración* (48) officially implemented between 2014 and 2018 – includes in its strategy 4.3. “Facilitate and promote integral health” the line of action 4.3.4. “Promote intercultural and linguistic attention to migrant people in health, gender, reproduction and human rights.” This perspective is particularly relevant in a country that is so socio-culturally and ethnically diverse^4^.

For this reason, the Ministry of Health has published the “Intercultural Guidelines for Health Service Personnel” (49) inviting to “*respect the culture*” of the patient and to “*distinguish the characteristics and cultural identities of each patient (…) to improve the quality of care*” (Third Guideline) and maintain an “*assertive communication* […] *regardless of whether there is communion with their beliefs or not*” (Eighth Guideline). Health personnel is also asked to adopt “*respect and dignified treatment* [as] *a fundamental norm*” (Fourth Guideline).

On the one hand, it is requested to “*avoid judgments and prejudices regarding the causes of the disease*” (Fifth Guideline) and only oppose the patient's actions if one is “*certain that it is a harmful practice*” (Sixth Guideline). We observe the construction of a discourse that denies any differentiation between migrant and local patients (following the principle of equality and non-discrimination), but at the same time a legitimization of differentiated care (by the intercultural perspective and respect for the culture of each person). On the other hand, they are forewarned that it is forbidden to execute “activities for which they are not trained or qualified” (Tenth Guideline); so, the other guidelines could be called into question. Faced with these seemingly contradictory instructions, it is understandable that practitioners who lack confidence in their ability to integrate these guidelines – and in their intercultural competence in general – strive to stay within the praxeogram^5^ and their “comfort zone” when meeting migrants.

As a matter of fact, in very few minutes, the staff must assess the patient's culture; in a context in which several declare doubt of their intercultural competence. Certainly, health professionals put the precariousness of their working conditions as a strong limitation to the application of interculturality in daily care: “*I don't see how we can have the time to meet a patient if we have an average of 7 minutes to attend them*” (general practitioner, public hospital). A doctor at a CSU delved into this before acknowledging that he simply didn't feel prepared to do so: “*Not only do we not have time and are saturated, but honestly, I'm not sure I have the necessary capacity*. […] *I can guarantee you the same quality in technical gestures, but we are not experts in interculturality*.” He continues: “*At school, we do not take classes in interculturality, only in medical ethics, and that is because it was in* [a renowned university] […] *I knew from this until now that I work for* [name of the institution of belonging].”

Similarly, a nurse confirmed that it was difficult for her to adapt to practice in a large public hospital because she studied in the nursing school of a private university and therefore did most of her practicums in the clinic – also private – with which this university has its agreements. She points out that “*cultural competency is not something you learn in school*”. Adding that she believes that healthcare professionals “*sometimes*” tend to “*use stereotypes*”.

In this way, we found that the simultaneous application of the principle of equal treatment and that of respect for the patient's culture causes misunderstandings and even internal conflicts in health providers who wonder how to treat everyone equally but at the same time adapt their practice to the individual characteristics of each patient.

Prud'homme (51) exposes the uncomfortable tension between the principles of equal treatment and individualization of care for caregivers:

While claiming a professional practice based on the principle of equal treatment, caregivers proceed daily to racially categorize the patients they receive. Yet, to “humanize care,” hospital professionals have, in recent years, also been encouraged to adapt their professional practice to the specificities – particularly the “cultural” ones – of each patient. (p. 85)

This situation inevitably raises the question of the “cultural competence”^6^ of healthcare workers. Indeed, migrants' understanding of their medical situation, the adoption of effective preventive measures, the implementation of health-oriented follow-up rather than symptom management are linked to agents' ability to understand and integrate undocumented migrants' cultural histories and perspectives into the therapeutic process. Conversely, the absence of “intercultural competence” can lead directly to a reinforcement of exclusionary logics and practices. So then, do medical personnel in Nuevo Leon have the tools to comply with the above-mentioned and seemingly contradictory “Intercultural Guidelines for Health Service Personnel”? The speeches analyzed below point to a divided response.

We were able to see a particular disparity between discourse and reality in a private hospital that treats “*between 5 and 6 migrants per month*”, according to an administrative official, although he explains that to “*not discriminate, we do not keep any records*”. He also confirms that all hospital workforces follow the interculturality courses taught by the State Health Secretariat: “*of course we do, we follow them*.” In a subsequent interview at the same hospital, the person responsible for the nursing department corroborates with a similar tone: “*of course, we receive updates*”, but confusing “interculturality” with “humanity” (“*we receive mostly courses in humanity*”). This person only gave a general explanation of how he applies the apprenticeship of these courses in his daily practice: “*we treat the outsider patients kindly and* [try] *to be human with them*”.

The same situation occurs in a CSU, where the social work manager recognizes that it is “*a new reality*” but declares to be “*qualified*” for having followed a “*3-hour course* [that] *teaches us about rights and interculturality* […] *and its importance*”. When asked about the existence of instructional materials that help him apply the knowledge in his daily activity, he answers: “*yes, there are materials* […] *on human rights, vulnerable populations, and interculturality*”. He explains that “*there are people at the national level who know and permeate us later*”. Who? “*A human rights chief* ” without further precision. Then, when asking if he has instructional materials in his possession, he showed a small calendar of the *Vete sano, regresa sano* program^7^ from 2013 – even though this program does not relate to foreign migrants in Mexico – which includes some brief recommendations on the side of the 12 months. In the end, the person qualifies his speech by claiming that his knowledge is based more on practice, due to its CSU location near a shelter for migrants, it receives between 4 and 5 of them daily.

Another general practitioner describes the courses taught by the public hospital where he works: “[It is] *an internal course that elaborates the teaching and training department for curricular validity and they build the course and see who can teach it*”. He becomes more critical when he stresses that “*these courses are valueless*”. In his opinion, the Ministry of Health courses “*serve for the certification accreditations of health centers*. […] *That's the real goal of the courses*”. The same impression on another CSU doctor: “*The problem is that there is no follow-up*. […] *It is very irregular. There is no evaluation of medical practices*”. These courses would therefore serve rather as an alibi to meet international requirements and to protect health facilities from recrimination.

The picture then looks not encouraging: although sometimes they offer a correct understanding of interculturality (“[the process by which] *we must take into consideration how the patient wants to be cared for, and not how the doctors believe that it should be*”, according to a general practitioner at a CSU), and the concern of health professionals to “humanize” and not discriminate is indisputable, the conditions under which they are asked to consider the diversity of their patients, combined with their insufficient preparation in intercultural circumstances, it seems to compromise the accomplishment of this objective.

In terms of continuing education, health authorities have put in place what Fortin and Laprise (53) call “*a system of ‘cultural understanding' in the hope of maximizing the reach and effectiveness of care*” (p. 202), but the evidence shows that these efforts are both sporadic and ineffective.

A social work manager explains how these trainings offered by the Ministry of Health work:

We were invited to take an online course [on interculturality] […] which was not mandatory […]. For face-to-face training, we have to organize ourselves because the services cannot remain deserted, we organize ourselves to see who can go and who stays. In fact, last year the “Medical Days” [of training] and their “Social Work Days” were about interculturality. All the themes were related to that, so a lot of people from other hospitals went.

This testimony indicates that training efforts are both sporadic, poorly organized, and attended by workers more out of obligation than to create a dynamic for improving care for users. A public health director acknowledges that not all these trainings are effective, although he tries to mitigate the reality:

We know that there are people who receive these pieces of training who have a mental square, in the sense that they don't know how to do it and how to receive it. Basically, the lack of knowledge, the lack of knowing how to solve [the new situations] means that they [the migrants] get a negative response from them [a refusal of care].

This is partly explained by the success indicators of these pieces of training, which are all numerical and “process” indicators (number of conferences given, number of participants in workshops, etc.), while no real measurement is made of a possible change in agents' representations. Nor are they supported in a possible transition toward an improvement of their daily practice. The doctor who provided us with the didactic materials for the Ministry of Health courses confirms this explanation. He states that he perceives them as an imposed constraint and that few of his colleagues experience these moments as an opportunity to improve their daily practice. He points out that the “training” consists of sitting and listening to a speaker who is a specialist in the topic of the day. The real contributions are therefore minimal.

Finally, one of these “specialists,” the Director of Human Rights Promotion at the Nuevo Leon State Human Rights Commission, explains along the same lines. To begin with, he states that these pieces of training “*in the health sector are not very effective, because there is no evaluation. In general, they are done in the form of a conference*”. He also indirectly confirms that these pieces of training are provided at least in part “*out of obligation*”: “*they opened the auditorium* [where the training takes place] *to us* (…) *because it was a training that was derived from a Recommendation* [issued by his institution in response to an observed violation of a human right].”

In fact, “interculturality” is understood by each person as they see fit. During the interviews, respondents defined it by saying that one must “*always take the patient's culture into account*” (general practitioner), “*take a step toward the other's culture, to understand him better*” (nurse), or “*know that migrants have different beliefs and habits from those of people here*”, but that one must “*respect them as they are*” (social worker).

The person in charge of nursing in a hospital does not hesitate to use the term “*illegal*” to refer to migrants (“*we want to help them, even if they are illegal*”), showing that behind good intentions there are perceptions (and actions) that may negatively impact their care-seeking process.

The common use of culturalism leads to a process of “essentialization” of migrants. A standardizing discourse separates users into categories, and migrants are then considered as a uniform “collective body” (regardless of their age, psychic state, gender, migratory trajectory, experiences, and health beliefs). Adopting this excessive generalizing attitude, a psychologist refers to the beliefs of migrants: “[the people] *of Honduras and El Salvador, or well the vast majority of the community in these countries, don't believe in psychology*.”

The “culture” of migrants is perceived as fixist, monolithic (that's why it is always cited in the singular), and linked to their nationality. Its determinism would have the value of evidence and causal explanation of health behaviors. Personal characteristics are erased by this “culture of origin,” which is necessarily perceived as an obstacle to care and must be modified. Even if the use of labels such as nationality or ethnicity to describe epidemics creates false relationships between these categories and diseases (54), a similar phenomenon has also been documented in other latitudes (15, 21, 55).

The recurrent use of the terms “migrants,” “these people,” “they,” “these populations” keep them at a distance as if they were a monolithic social group with homogeneous and stable cultural attributes of their own, and different from the majority and dominant group. They are recognized more for their supposed differences than for their real identity. It got to an extreme where a social worker who alluded to the name “*the Hondurans*” to refer to migrants in general.

The confinement of undocumented migrants in the labels of “migrants,” “Central Americans,” or “Hondurans” in the latter case, serves as a cognitive device of “identity assignment.” It simplifies the understanding of a complex social situation and immediately places the caregiver in a much more restricted “intervention framework,” which allows him to avoid awkward questioning. As Austin explains, “*saying is doing*”: language is performative in that it can create the situations it states Austin 1970, cited by Musso (56). The anxiety produced by the appearance of an atypical user is then tempered by the label applied, which allows the interlocutor of the “migrant” to use his ordinary “toolbox” and minimize the embarrassment caused.

In sum, if the reality of the universality of care is in doubt, the reality of othering is undeniable. *Inter-*culturality is understood in fact as an *alter-*culturality, where the migrant is “the different” to the “reference” culture of the majority population (without importing the ultra-simplification of this conception, as if there were only two homogeneous cultures in contact).

## The Use Of Stereotypes and The Naturalization Of Differences In Nuevo Leon

The foregoing leaves room for the generalization of ethnic stereotypes that essentialize migrants, while blaming them for their health conditions. Hence, if the working conditions of health professionals complicate the application of their already substandard intercultural competence, what cultural references do they use to effectively incorporate the culture of migrants into the healthcare process, as postulated by the intercultural perspective? Everything indicates the only references available are those that prevail in the collective imagination in the state of Nuevo Leon, although they correspond more to a set of stereotypes^8^ about migrants than to social reality.

### Current Stereotypes

To understand the complexity of barriers in access to health, it is essential to consider the values, prejudices, and stereotypes that determine the social perception of migrants. In Nuevo Leon, the “ethnic” and “cultural” stereotypes in force predispose the caregivers. In this regard, De Rudder and Vourc'h (57) state that:

This stereotypical construction may seem caricatural. It is clearly not at work permanently and in its entirety, but it produces a coherent picture of the new dangerous class. It provides an available stock of interpretations of social reality, stigmatization that involves all the protagonists of the relationship, the denouncers as well as the denounced and their defenders. (p. 11)

On its side, John Dovidio defines prejudice and stereotypes as intrapsychic phenomena. Therefore, the level of awareness of the person who produces them may vary (58). They are related to ignorance, and they are activated unconsciously. Dovidio distinguishes prejudice from stereotyping, the former corresponding to an unverifiable negative attitude toward a group or individual, while the latter is a generalized belief in relation to the assumed characteristics of a group (59). Both can be deeply ingrained and even the provision of authentic and verifiable information may fail to change them.

Depending on the circumstances, the specific attributes on which stereotypes are based can be transformed into stigmas. In this regard, it is difficult not to refer to Erving Goffman, who described stigma as a phenomenon whereby an individual may be discredited and rejected by society because of a specific attribute, behavior, or reputation (60). Goffman views stigma as a process by which the reaction of others ruins the individual's identity, which then becomes undesirable. He argues that stigma is intimately associated with stereotyping and that both are linked to unconscious expectations and norms that act as invisible arbiters in all social encounters. However, the stigmatizing attribute is not discrediting in and of itself or for anyone who possesses it; it depends on the specific situation of the possessor.

In the case of migrants, their stigmas seem instead to be very entrenched and almost impossible to eliminate, the first of which is their irregular situation, which aggravates the way they are looked at (61). They would be victims of a kind of concentration of negative attributes that would place them in a deeply discredited category: that of lazy, dirty and with bad manners.

The healthcare workers therefore have normative expectations of foreigners even before interacting with them, and consequently expect them to act in accordance with these expectations. These “demands” and attributed characters constitute a “virtual social identity,” distinct from the real one. The host society then explicitly assumes to require foreigners to conform to their preconceived expectations (for example, accepting migrants only if they comply with what is expected from them). Leyva et al. illustrated this in their research on the stigmatization of migrants in relation to diseases (62). The authors show that populations in the Mexico-U.S. border area identify migrants as carriers of HIV-AIDS, as well as other infections such as dengue, malaria, tuberculosis, and chikungunya, when the available scientific evidence tends to show that this is not the case.

In short, the theorization of otherness in the name of the “culture” of the “others” is operational to explain the behaviors and legitimize the postures of each intervener. It also supports the “biologization” of difference in clinical relations through the supposed or real origins of migrants. Differences, especially social ones, are then “naturally” instituted. Above all, this recourse to culture prevents caregivers from questioning their attitudes and behaviors by constantly offering them “ready-made” interpretations that justify their behaviors.

Indeed, the fieldwork highlighted numerous assumptions about migrants' beliefs, experiences, and health practices based on impressions – usually negative – or in observations and particular experiences of the interviewees: “*They are people who arrive drunk, drink too much, and sometimes get stoned and fight*”, enunciates a nurse at a private hospital; while the person in charge of social work at a public hospital explains that “*it's difficult to trust them because they lie considerably* [even if they do] *out of fear and to protect themselves*”. He continues: “*They are very distrustful and therefore aggressive*. […] *could tell you they're rude* […] *it's becoming a problem* […] *they're not productive, do you understand what I mean?*”. Finally, a psychologist who claims to attend in “*emergency containment*” to migrants, explains that “*these people do not want medical advice* […] *they just want you to give them a pill*” (author's emphasis).

This type of stereotyping can produce discrimination, racism, and sexism (58), as when a general practitioner in a CSU derides migrants by referring to their supposedly habitual use of “*healers*” in an ironic tone and laughter, even though he admits that he does not know them well, as “*they only come here very sporadically*”.

The most discriminatory and racist-tinged comments were from a nurse at a private hospital, when he claimed that “*some are very brown* [of black skin, author's note], *so we assume they are not from here*”^9^, before continuing: “*We are equals, but we are not the same, do you understand me?*”, referring to a Honduran person who attended briefly, and immediately clarifying his comment: “*I am not racist, quite the opposite* […] [but] *they're from different cultures*”.

Even those who claim to have accurate knowledge of the migrants' health needs, as an administrative manager of a hospital, who does not hesitate to affirm that “*we all know the reasons of their vulnerability*”, usually proceed to a culturalist assessment of the health situation of migrants. The same person explains later: “*We need to find some compatibility between Western medicine and the traditional health practices they may have*” (author's emphasis).

This testimony perfectly underlines how the alleged “vulnerability” of migrants is almost systematically related to cultural factors and not social factors; although it has long been known that health outcomes of mobile populations are primarily defined by their social and economic conditions, above cultural and individual issues (59, 64). In the same way, strong socioeconomic constraints, discrimination in care, and the stigma of poverty play a much larger role in their poor health outcomes than “cultural” issues (62, 64–66).

In Nuevo León, an “ethnic category” of the “Central American migrant” is being forged, based on supposed cultural practices related to their culture, composed of supposed habits, customs, mores or ways of living distinct from those of the local population. The Other then embodies the difference, which can intrigue and whet curiosity, but also inspire fear. The production of discrimination against migrant populations highlights the social construction of these “differences,” which raise the question of otherness, and whose main manifestations have been presented here.

The representations and imaginaries in force in Nuevo León do not fail to create tensions, which revolve around the perceived differences, and lead to serious consequences on their access to healthcare. Indeed, this type of stigmatization by culture opens the door to differential treatment compared to other non-migrant users, and some individuals may be victims of mistreatment and even physical or verbal violence. In the clinical field, this can result in inadequate treatment, based on incorrect representations of the migrant patient's ability to follow a treatment, or in the refusal of their decisions, even though they are made in an autonomous, free, and informed manner.

It is worth noting that, from the 21 completed interviews with health service professionals, only one person forcefully dismissed cultural factors as the primary explanation for migrants' penurious access to health: “*health is universal and should not be denied to anyone, regardless of their origin. Unfortunately, they are discriminated against for false beliefs. They are denied attention, but cultural issues don't have much to do with it*” (director, CSU).

### The Naturalization of Differences

This ordinary culturalist interpretation by healthcare providers not only serves to conceal possible discrimination, but also contributes to its production, by making migrants exclusively responsible for their difficulties in accessing healthcare, and by “naturalizing” the barriers to health. They *are* the risk factors, not the situations in which they find themselves (whereas anyone in their social circumstances would probably proceed in the same way). By doing so, the real specificities and vulnerabilities of migrants are obscured.

In addition, in some cases essentialization and culturalism concur with blaming migrants for their health problems: “*with migrants, we try to be flexible*”, although “*they do not know how to express their ills*” (*Seguro Popular* Administrative agent). In this perspective, if they do not use the public health services to which they are entitled, it would be a consequence of their lack of adaptation or communication.

A nurse of a large hospital argues that “*we speak the same language, but they don't understand us*” to justify that if migrants do not follow precisely the instructions given by the medical staff, it is because of habits specific to their culture, because, in his opinion, the health personnel always explain in detail the instructions to follow.

This accentuates the penurious understanding of interculturality by health personnel, and scarcely any claim to reflect on their practice or cultural identity. The foreigner, the one who comes from another place, is the one who must adapt to the system as it preexists, which at the same time denies responsibility and exempts the health institution from any reflection on its role in the production of differences; as mentioned by this nurse who never questions the reasons for the low adherence of certain migrants to treatments: “*Although their state of health is worrying, they stop attending medical consultations, or leave the programs*. […] *Then some come back* […] *but they don't understand that it's not good to act like that*”.

Finally, as an extreme case, we look at the situation of a foreign patient who attended a private hospital to dismiss possible tuberculosis, a disease whose determinants are social (living conditions, health habits, immunological conditions, among others). The doctor who treated him gave only minimal explanations about the disease, and mainly asked him to undergo a series of clinical tests; justifying a posteriori the absence of explanation with a foul “*whatever, they will not understand*” (In clear contravention to the Ninth Guideline: “Clearly explain the disease, treatment, and care, making sure that users and their families understand you, considering their age, sex, ethnic group, language, sexual preference, religion, disability and illness”).

Naturally, despite potentially having a serious illness, the foreign patient left the hospital without the capacity to explain the meaning of the clinical tests requested. In this case, the doctor's prejudice became “self-realizing,” because they did not encourage the patient to be involved in their healing through a correct explanation, so it decreased his possibility of understanding the instructions.

## Conclusion

The collected testimonies show a strong tendency in administrative and health personnel to proceed – usually unconsciously – to an “essentialization” of foreigners in a mobility situation in Nuevo Leon. The medical world is far from cultural detachment and the national political context. The identified differences are widespread, and culture becomes a global explanation. Such categorization is primarily based on stereotypes present in the dominant culture of their society of belonging and in their experiences.

This “culturalist” explanation of everything that is not immediately understandable singles out the migrant population because of the differences that supposedly characterize it. Excessive recourse to “culturalism” to explain the behavior of migrants can influence the attention given to them, as well as in their adherence to treatments; and therefore, harm their health. It reinforces the conditions of structural vulnerability that migrant populations face.

Migration acts as a disruptive element in the care system. Why does the issue of migrants challenge and destabilize health professionals so much? They are destabilized by this “elsewhere.” The deficient intercultural competence recognized by several of the same agents in the health system doesn't allow them to comply with the instructions given by health authorities in this area. These remain as mere pronouncements that will hardly exceed the level of good intentions and whose fulfillment will depend on the level of empathy that animates each agent. Then, migration forces caregivers to reinvent their methods of care and rethink their actions.

Resorting to “cultural” explanation avoids reflecting on practical obstacles in accessing health services: lack of information for migrants on their options to access the health system, complex and expensive physical access to healthcare facilities, and complicated administrative path to follow to get the desired attention. All this in a context of othering encouraged by the generalization of negative – though often unintentional – stereotypes about migrants.

Indeed, it is very complicated for a person who has just arrived in Mexico and who does not know the functioning of the health system, to register in the *Seguro Popular* if its existence is unknown; to request a letter of reference at a CSU to demand an appointment with a specialist in a hospital in case you require one; to visit the same hospital to get a “letter of reference” necessary to get an appointment, among other requirements to follow. All the above, far from being due to cultural factors, can take weeks or months in a context of fear of reporting and rejection or denial of care for lack of a valid immigration document.

For all these reasons, the health system *itself* could be a stressor. Reasoning through “the culture” thus reifies behaviors and allows for the depoliticization of the real social issues (the precarious living conditions and suffering from persecution imposed on migrants in Mexico), by making them bear the burden of the obstacles to health they face. Therefore, any effort to improve both the health status of undocumented migrants and their access to care must devise mechanisms to invite health institutions to stop shirking their responsibilities. Paradoxically, the “invisibles” (migrants) may well be useful in making visible the hidden problems of the Mexican health system.

## Data Availability Statement

The raw data supporting the conclusions of this article will be made available by the authors, without undue reservation.

## Ethics Statement

The studies involving human participants were reviewed and approved by Universidad de Monterrey. Written informed consent for participation was not required for this study in accordance with the national legislation and the institutional requirements.

## Author Contributions

The author confirms being the sole contributor of this work and has approved it for publication.

## Conflict of Interest

The author declares that the research was conducted in the absence of any commercial or financial relationships that could be construed as a potential conflict of interest.

## Publisher's Note

All claims expressed in this article are solely those of the authors and do not necessarily represent those of their affiliated organizations, or those of the publisher, the editors and the reviewers. Any product that may be evaluated in this article, or claim that may be made by its manufacturer, is not guaranteed or endorsed by the publisher.

## Footnotes

^1^As the Royal Spanish Academy points out, the notion of “culture” is subject to debate. In the present work, we understand it as a system of interconnected beliefs and customs to create a way of life and knowledge.

^2^“Culturalism” consists of reducing a minority population (Central American migrants in this case) to their cultural otherness, and therefore define it mainly by its origin, using its cultural characteristics - real or assigned - to explain all or most of their behavior.

^3^To address growing health inequalities, a protection mechanism has been set up in 2003 under the name of *Seguro Popular* [“Popular Insurance”]. This means of public financing of basic health needs is oriented toward the population deprived of any social coverage. At the end of 2014, the *Seguro Popular* was opened to all persons present in Mexican territory, including undocumented foreigners; officially allowing them access to a system that was previously forbidden to them (apart from emergency medicine).

^4^Indigenous people represent about 15% of the population and there are 68 national languages.

^5^Also called “interaction schema”, Moirand explains that it is a “*schema of ideal, verbal or gestural actions which is the basis of the course of daily or professional activities*” (50) (p. 92). This term would be more appropriate than “*script*” or “*scenario*,” which are preferred “*when it comes to strictly verbal activities*” (50).

^6^Understood as “an ability to understand and effectively communicate with people across cultures and it includes: awareness of one's own cultural beliefs, attitude toward different cultures, knowledge of different cultural practices, and cross-cultural skills” (52) (p. 207). “Cultural” competence is not equivalent to “intercultural” competence, the latter includes a communicative (verbal and non-verbal), cognitive (knowledge of the cultures present) and affective (sensitive understanding of the other) dimension. It allows to understand similarities and differences and to establish a relationship with the foreign patient.

^7^This answer is astounding for four reasons: first, this bi-national Mexico-U.S. program set up in the 2000s has never been fully implemented. Most importantly, it is not at all about foreign migrants in Mexico, but about Mexicans going to the United States. Second, the fact that the social worker considered a desk calendar as “teaching material” to develop a job skill. Finally, the date: the interview took place in 2017 and the “teaching material” was from 2013.

^8^We refer here to the stereotype in its classical acceptance enunciated by Lippman (26), according to which the production of simplifying images allows to interpret and adapt with greater confidence to the complexity of the social environment; although in our case study, it leads to a separate categorization of migrant patients.

^9^This person completely ignores what is now frequently referred to as Mexico's “third root”: the populations of African descent. The latter represent 1.4 million people (63). Despite their numbers, their existence has been denied until recently. Thus, the category “Afro-Mexican” only appeared in population censuses as of 2020.

## References

[1] Paz M Cerda A Ledón A. Mirar las fronteras desde el sur: Salud y migración en la frontera México-Centroamérica. Distrito Federal, México: UAM. (2016).

[2] FitzGerald C Hurst S. Implicit bias in healthcare professionals: a systematic review. BMC Med Ethics. (2017) 18:19. 10.1186/s12910-017-0179-8 28249596 PMC5333436

[3] Leyva R Infante C Serván-Mori E Quintino F Silverman O. Acceso a servicios de salud para los migrantes centroamericanos en tránsito por México. CANAMID Policy Brief Series, PB05, México: CIESAS (2015). Available online at: https://bit.ly/3tZJBzm (accessed February 10, 2022).

[4] Ransing R Ramalho R de Filippis R Ojeahere MI Karaliuniene R Orsolini L. Infectious disease outbreak related stigma and discrimination during the COVID-19 pandemic: Drivers, facilitators, manifestations, and outcomes across the world. Brain Behav Immun. (2020) 89: 555–8. 10.1016/j.bbi.2020.07.033 32731007 PMC7384410

[5] Von Unger H Scott P Odukoya D. Constructing immigrants and ethnic minority groups as “carriers of disease”: Power effects of categorization practices in tuberculosis health reporting in the UK and Germany. Ethnicities. (2019) 19:518–34. 10.1177/1468796819833426

[6] Ulaby N. Little Demons, Death Biting Dogs: How We Picture Disease. NPR (2020). Available online at: https://www.kcrw.com/news/shows/npr/nprstory/823949176 (accessed January 25, 2022).

[7] Place V Nabb B Viksten Assel K Bäärnhielm S Dalman C Hollander AC. Interventions to increase migrants' care-seeking behaviour for stigmatised conditions: a scoping review. Soc Psychiatry Psychiatr Epidemiol. (2021) 56: 913–30. 10.1007/s00127-021-02065-1 33778914 PMC8192321

[8] Gómez L Gómez LA. Un siglo después de la “gripe española”: contribución de la Gran Guerra y conocimiento del genoma como herramienta para control de la influenza. Biomédica. (2019) 39:17–21. 10.7705/biomedica.v39i1.4884 31021543

[9] Ochieng BMN. Black African migrants: the barriers with accessing and utilizing health promotion services in the UK. Eur J Public Health. (2013) 23:265–9. 10.1093/eurpub/cks063 22683768

[10] Riggs E Davis E Gibbs L Block K Szwarc J Casey S. Accessing maternal and child health services in Melbourne, Australia: Reflections from refugee families and service providers. BMC Health Serv Res. (2012) 12:117. 10.1186/1472-6963-12-117 22587587 PMC3424108

[11] Ministry of Health [Secretaría de Salud]. ¿Qué hacemos? (n.d.). Available online at: https://www.gob.mx/salud/que-hacemos (accessed May 21, 2022).

[12] Pretceille M. L'interculturel comme paradigme de transgression par rapport au culturalisme. Voix Plurielles. (2015) 12:251–63. 10.26522/vp.v12i2.1286

[13] Fassin D. Repenser les enjeux de santé autour de l'immigration. Hommes et Migrations. (2000) 1225:5–12. 10.3406/homig.2000.3506

[14] Porcher L. Parcours de l'interculturalité, BNF: Actes du Colloque. Chemins d'accès: Les nouveaux visages de l'interculturalité. (2004). p. 4–6. Available online at: https://plone.unige.ch/art-adr/cases-affaires/affaire-manuscrits-coreens-2013-france-et-coree-du-sud-1/bnf-actes-du-colloque-chemins-dacces-les-nouveaux-visages-de-linterculturalite-18-novembre-2004/at_download/file

[15] Cognet M Montgomery C. É*thique de l'altérité: La question de la culture dans le champ de la santé et des services sociaux*. Québec: Presses de l'Université Laval. (2007).

[16] Cognet M Bascougnano S Adam-Vezina E. Traitement différentiel dans les parcours thérapeutiques. [Rapport de recherche] DREES-Mire. (2009). Available online at: https://hal-univ-paris.archives-ouvertes.fr/hal-00760137 (accessed January 25, 2022).

[17] Cognet M. La vulnérabilité des immigrés : Analyse d'une construction sociale. In: Saillant F Clément M Gaucher C editors. Identités Vulnérabilité, Communautés. Montreal, QC: Éditions Nota Bene (2004). p. 155–188.

[18] Cognet M. Les représentations des praticiens vis-à-vis de l'ethnicité dans la rencontre clinique, L'immigration et l'intégration au cœur des débats : recherches, politiques et pratiques. Montréal, QC: 7éme Conférence Nationale Metropolis (2004).

[19] Fassin D. L'internationalisation de la santé entre culturalisme et universalisme. Esprit (1997) 229:83-105.

[20] Roy B. Sang sucré, pouvoirs codés, médecine amére. Diabéte et processus de construction identitaire: les dimensions socio-politiques du diabétes chez les Innus de Pessant. Montreal, QC: Presses de l'Université Laval (2003). p. 247.

[21] Fortin S. Conflits et reconnaissance dans l'espace social de la clinique: les pratiques cliniques en contexte pluraliste. Anthropol Soc. (2013) 37:179–200. 10.7202/1024085ar

[22] Roy B. Vulnérabilité des autochtones ou regard tutélaire des milieux de la santé. In: Saillant F Clément M Gaucher C editors. Identités Vulnérabilité, Communautés. Montreal, QC: Éditions Nota Bene (2004). p. 189–229.

[23] Cognet M. Migrations, groupes d'origines et trajectoires : vers une ethnicisation des rapports socioprofessionnels ? Villeneuve d'Ascq: Presses Universitaires du Septentrion (1999). p. 368.

[24] Ho MJ. Sociocultural aspects of tuberculosis: a literature review and a case study of immigrant tuberculosis. Soc Sci Med. (2004) 59:753–62. 10.1016/j.socscimed.2003.11.033 15177832

[25] Witzig R. The medicalization of race: scientific legitimization of a flawed social construct. Ann Intern Med. (1996) 125:675–9. 8849153 10.7326/0003-4819-125-8-199610150-00008

[26] Lippman W. Public Opinion. New York, NY: Harcourt, Brace and Company (1922).

[27] Comisión Interamericana de Derechos Humanos Organización de Estados Americanos. Derechos humanos de los migrantes y otras personas en el contexto de la movilidad humana en México. (2013). Available online at: https://bit.ly/3Cj9Nrm (accessed January 24, 2022).

[28] WHO South-East Asia Region. Health of refugees and migrants. Practices in Addressing the Health Needs of Refugees and Migrants. (2018). Available online at: https://www.who.int/publications/i/item/health-of-refugees-and-migrants—practices-in-addressing-the-health-needs-of-refugees-and-migrants-who-south-east-asia-region (accessed January 25, 2022).

[29] Global Knowledge Partnership on Migration Development (KNOMAD). Human Rights Indicators for Migrants and their Families. (2015). Available online at: https://www.ohchr.org/Documents/Issues/Migration/Indicators/WP5_en.pdf (accessed May 20, 2022).

[30] IFRC. Common Barriers to Healthcare Encountered by Migrants. Least Protected, Most Affected: Migrants and Refugees Facing Extraordinary Risks During the COVID-19 Pandemic. (2020). Available online at: https://reliefweb.int/sites/reliefweb.int/files/resources/IFRC-report-COVID19-migrants-least-protected-most-affected.pdf (accessed May 21, 2022).

[31] Organisation internationale pour les migrations. Migration internationale, santé et droits de l'homme. (2013). Available online at: https://www.ohchr.org/Documents/Issues/Migration/WHO_IOM_UNOHCHRPublicationFR.pdf (accessed January 24, 2022).

[32] Holmes S. The clinical gaze in the practice of migrant health: Mexican migrants in the United States. Soc Sci Med. (2012) 74:873–81. 10.1016/j.socscimed.2011.06.067 21992736

[33] Good D James C Good BJ Becker AE. The culture of medicine and racial, ethnic, and class disparities in healthcare. Confronting Racial and Ethnic Disparities in Health Care. Washington, DC: Institute of Medicine – National Academies Press. (2003).

[34] Arce IO. La formación del profesional en salud y la incorporación de la Interculturalidad en la Currícula Facultativa. Gac Méd Bol. (2013) 36:48–50. Available online at: http://www.scielo.org.bo/scielo.php?script=sci_arttext&pid=S1012-29662013000100012

[35] Parsons T. The Social System. Glencoe, IL: The Free Press. (1951).

[36] Mechanic D. The concept of illness behavior. J Clin Epidemiol. (1962) 15:189–94. 10.1016/0021-9681(62)90068-1 14471950

[37] Zola IK. Culture and symptoms—an analysis of patient's presenting complaints. Am Sociol Rev. (1966) 31:615–30. 10.2307/2091854 5977389

[38] Paul B. The role of beliefs and customs in sanitation programs. Am J Public Health Nation's Health. (1958) 48:1505–6. 10.2105/AJPH.48.11_Pt_1.1502 13595147 PMC1551810

[39] Lebreuilly R Sakkour S Lebreuilly J. L'influence de la culture dans l'expression verbale de la douleur: étude comparative entre des patients cancéreux français et syriens. Rev Int Soins Palliatifs. (2012) 27:125–9. 10.3917/inka.124.0125 23548892

[40] Alarcón AM Vidal A Neira J. Salud intercultural: elementos para la construcción de sus bases conceptuales. Rev Méd Chile. (2003) 131:1061–5. 10.4067/S0034-98872003000900014 14635595

[41] Anderson LM Scrimshaw SC Fullilove MT Fielding JE Normand J. Culturally competent healthcare systems: a systematic review. Am J Prev Med. (2003) 24:68–79. 10.1016/S0749-3797(02)00657-8 12668199

[42] Brach C Fraserirector I. Can cultural competency reduce racial and ethnic health disparities? A review and conceptual model. Med Care Res Rev. (2000) 57:181–217. 10.1177/1077558700057001S09 11092163 PMC5091811

[43] Betancourt JR Green AR Carrillo JE Ananeh-Firempong O II. Defining cultural competence: a practical framework for addressing racial/ethnic disparities in health and health care. Public Health Rep. (2003) 118:293–302. 10.1016/S0033-3549(04)50253-4 12815076 PMC1497553

[44] Dauvrin M Lorant V. Adaptation of health care for migrants: whose responsibility? BMC Health Serv Res. (2014) 14:294. 10.1186/1472-6963-14-294 25005021 PMC4108228

[45] Bernales M Pedrero V Obach A Pérez C. Competencia cultural en salud: una necesidad urgente en trabajadores de la salud. Rev Méd Chile. (2015) 143:401–2. 10.4067/S0034-98872015000300018 26005830

[46] Antezana AM Osmar I. La formación del profesional en salud y la incorporación de la Interculturalidad en la Currícula Facultativa. (2013). Available online at: https://www.semanticscholar.org/paper/La-formación-del-profesional-en-salud-y-la-de-la-en-Antezana-Osmar/34a7bf561d5529593752a91fa856a4d8fa9c5e6b (accessed May 30, 2022).

[47] Spector RE. Cultural diversity in health and illness. J Transcult Nurs. (2002) 12:197–9. 10.1177/10459602013003007 12113150

[48] Instituto Nacional de Migración. Programa Especial de Migración (PEM) 2014-2018. (2016). Available online at: https://www.gob.mx/inm/documentos/programa-especial-de-migracion-pem-2014-2018-18281 (accessed January 24, 2022).

[49] Secretaría de Salud. Lineamientos Interculturales Para el Personal de los Servicios de Salud. (2013). Available online at: https://bit.ly/3tPWoUI (accessed May 21, 2022).

[50] Moirand S. L'évaluation dans les discours scientifiques et professionnels. Les Carnets du Cediscor. (1995) 3:81–93. 10.4000/cediscor.497

[51] Prud'homme D. Du soin global au traitement discriminatoire: La prise en charge de patientes identifiées comme roms dans un service de gynéco-obstétrique parisien. Terrains Trav. (2016) 29:85–104. 10.3917/tt.029.0085

[52] Medina MS Maerten-Rivera J Zhao Y Henson B. A systematic review of assessment tools measuring cultural competence outcomes relevant to pharmacy education. Am J Pharm Edu. (2022) 86:207–17. 10.5688/ajpe8672 35027358 PMC10159442

[53] Fortin S Laprise E. É*thique de l'altérité: la question de la culture dans le champ de la santé et des services sociaux*. Québec: Les Presses de l'Université de Laval. (2007).

[54] Williams J González-Medina D Le Q. Infectious Diseases and Social Stigma. Infect Dis Soc Stigma. (2011) 7:2–14. 10.15208/mhsj.2011.127

[55] Fassin D Dozon J. Critique de la santé publique: Une approche anthropologique. Paris: Balland. (2001). p. 7–19.

[56] Musso S. Comment l'anthropologie de la santé éclaire: certains enjeux des migrations. Idées Econ Soc. (2017) 189:20–7. 10.3917/idee.189.0020

[57] De Rudder V Vourc'h F. Assignation et discrimination racistes: enquêtes dans le monde du travail en France. Divers Urbaine. (2008) 8:7–23. 10.7202/018614ar

[58] Dovidio JF. The SAGE Handbook of Prejudice, Stereotyping and Discrimination. (2013). United Kingdom: SAGE.

[59] Dovidio JF. The Social Psychology of Discrimination: Theory, Measurement and Consequences dans Making Equality Count. (2010). Ireland: Liffey.

[60] Goffman E. Stigma Notes on the Management of Spoiled Identity. USA: Prentice-Hall Inc. (1963)

[61] Schuster L Majidi N. Deportation Stigma and Re-migration. J Ethn Migr Stud. (2015) 41:635–652. 10.1080/1369183X.2014.957174

[62] Leyva R Charvel S Infante C. Migración, VIH y acceso a servicios de salud en México, México : Secretaría de Salud. (2018). Available online at: https://www.gob.mx/cms/uploads/attachment/file/498523/Migraci_n_y_VIH_2019_No14.pdf (accessed May 21, 2022).

[63] INEGI. Perfil sociodemográfico de la población afrodescendiente en México (2017). Available online at: https://bit.ly/3zonvHA (accessed January 25, 2022).

[64] Stoesslé P González F Santos J Sánchez N. Risk factors and current health-seeking patterns of migrants in northeastern mexico: healthcare needs for a socially vulnerable population. Front Public Health. (2015) 3:191. 10.3389/fpubh.2015.00191 26301213 PMC4526788

[65] Staszak JF. Other/ Otherness. International Encyclopedia of Human Geography. In Kitchin R Thrift N editorss. Amsterdam: Elsevier. (2009). p. 43–7.

[66] Moya J Uribe M. Migración y salud en México: una aproximación a las perspectivas de investigación; 1996–2006. Organización Panamericana de la Salud. (2007). Available online at: http://www.biblioteca.cij.gob.mx/Archivos/Materiales_de_consulta/Drogas_de_Abuso/Articulos/migracion45.pdf

